# Peripheral Blood-Derived Mesenchymal Stem Cells Modulate Macrophage Plasticity through the IL-10/STAT3 Pathway

**DOI:** 10.1155/2022/5181241

**Published:** 2022-04-11

**Authors:** Qi-Ming Pang, Rui Yang, Meng Zhang, Wang-Hui Zou, Nan-Nan Qian, Qi-Jing Xu, Hui Chen, Jia-Chen Peng, Xiao-Ping Luo, Qian Zhang, Tao Zhang

**Affiliations:** ^1^Key Laboratory of Cell Engineering of Guizhou Province and Regenerative Medicine Centre, Affiliated Hospital of Zunyi Medical University, Zunyi, Guizhou, China; ^2^Department of Orthopedics, Affiliated Hospital of Zunyi Medical University, Zunyi, Guizhou, China; ^3^Department of Human Anatomy, Zunyi Medical University, Zunyi, Guizhou, China

## Abstract

Mesenchymal stem cells (MSCs) are multipotent cells that can skew the balance of M1/M2 macrophage polarization towards the M2 phenotype via their paracrine effects, thereby promoting anatomical and functional recovery after many inflammatory diseases induced by macrophages. However, the underlying mechanism is still poorly understood. This study focused on the IL-10/STAT3 pathway and investigated whether IL-10 secreted by PBMSCs could mediate M2 polarization through the activation of this pathway. In this study, a Transwell system was used for coculturing macrophages and PBMSCs. ELISA and RT-qPCR analysis found that PBMSCs and their conditioned media (P-CM) significantly induced the expression of IL-10, while significantly inhibiting the expression of IL-1*β* and TNF-*α*; moreover, this effect could be reversed by adding Ab9969 (an IL-10 neutralizing antibody) and Stattic (a STAT3 inhibitor). Furthermore, western blotting and immunofluorescence assays demonstrated that JAK1/STAT3 signaling was significantly upregulated in macrophages cocultured with PBMSCs or P-CM, accompanied by an increase in the M2 biomarker CD206 and a decrease in the M1 biomarker CD86. This effect could also be reversed by blocking the IL-10/STAT3 pathway with Ab9969 and Stattic. In summary, PBMSCs could mediate the polarization of M2 macrophages by activating the IL-10/STAT3 pathway.

## 1. Introduction

As an important integral component of the innate immune system and cellular immunity, macrophages can not only maintain normal homeostasis but also play a critical role for repairing damaged tissues [1, 2]. In response to specific stimuli, macrophages can differentiate into multiple phenotypes with diverse functions [3]. “Classically activated” M1 macrophages, induced by lipopolysaccharide (LPS) and interferon- (IFN-) *γ*, are characterized by the production of substantial matrix metalloproteinases, reactive oxygen species, and proinflammatory cytokines (interleukin- (IL-) 1*β* and tumor necrosis factor- (TNF-) *α*), which enable the elimination of harmful pathogens and create a sterile microenvironment for tissue repair [4, 5]. In contrast, “alternatively activated” M2 macrophages facilitate tissue repair by secreting anti-inflammatory cytokines such as IL-10, which can also be regarded as a marker of M2 [6–9]. Studies have shown that unbalanced polarization with the excessive production of M1 macrophages is the pathogenesis of many inflammatory diseases, such as spinal cord injury (SCI), coronavirus disease, hypertension, diabetes, osteoarthritis, systemic lupus erythematosus, atherosclerosis, and inflammatory bowel disease [2, 10–12]. A shift from inflammatory macrophages towards the M2 phenotype has been definitively proven to inhibit the occurrence and evolution of related diseases [13–15].

Mesenchymal stem cells (MSCs) are characterized by adherent growth, specific surface markers, and multilineage differentiation [16]. Because of their strong immunomodulatory ability, MSCs have emerged as promising regenerative seed cells, with striking therapeutic effects in inflammatory disease models [17–20]. At present, MSCs can be isolated from a variety of adult tissues (e.g., peripheral blood, bone marrow, and adipose tissue) and neonatal tissues (e.g., amniotic membrane, placenta, and umbilical cord) [21, 22]. Despite the fact that MSCs from different sources have common effects in moderating the balance of immune cells in case of inflammatory diseases, their cytokine expression profiles, the strength of immune modulation, and the underlying mechanisms vary [23–26]. Consequently, it is necessary to study the regulatory roles of distinct MSCs on immune cells, together with their related mechanisms, before further application.

Apart from immunoregulatory properties, peripheral blood-derived mesenchymal stem cells (PBMSCs) have specific advantages in the ease of sample collection, as its collection causes less pain to patients compared with the collection of MSCs from other sources [27, 28]. Our previous study on PBMSCs showed that the transplantation of PBMSCs into rat models of SCI could effectively promote the recovery of hind limb motor function, which could be explained by the conversion of macrophages towards an M2 phenotype [29]. Subsequently, we established an indirect coculture system of macrophages and PBMSCs or bone marrow mesenchymal stem cells (BMMSCs). Consequently, we found that compared to BMMSCs, PBMSCs could induce M2 polarization more effectively, with a significant upregulation of IL-10 and downregulation of IL-1*β* and TNF-*α* in the supernatant [26]. Amongst these, the elevated levels of IL-10 piqued our interest.

IL-10 is an anti-inflammatory cytokine that plays a key role in driving the regulation of various anti-inflammatory processes [30]. This molecule combines with its receptors, IL-10R1 and IL-10R2, to activate Janus kinases (JAKs), which subsequently phosphorylate signal transducer and activator of transcription 3 (STAT3); this in turn can enter the nucleus and regulate the expression of related genes [31–33]. Recent studies have revealed that macrophages are the major target cells through which IL-10 exerts its anti-inflammatory effects, and the activation of the IL-10/STAT3 pathway results in macrophage polarization towards the M2 phenotype [34–36]. Moreover, numerous studies have documented that MSCs are able to secrete IL-10 [37]. Therefore, we hypothesized that IL-10 secreted by PBMSCs may induce a phenotypic shift of macrophages through the activation of the IL-10/STAT3 pathway.

In this study, we first measured the expression of M1/M2-related cytokines in macrophages in a coculture system and then investigated the relationship between the IL-10/STAT3 pathway and its characteristic cytokines using Ab9969 and Stattic. We further explored the molecular mechanisms by which PBMSCs mediate M1/M2 conversion, by analyzing the IL-10/STAT3 pathway, hoping to establish fundamentals for future applications.

## 2. Materials and Methods

### 2.1. Animals

Sprague Dawley (SD) rats (weight: 150–250 g; both sexes) were provided by the Animal Center of the Third Military Medical University (production license number: SCXK Yu 2017-0002). All animal experiments were performed in accordance with the 3Rs (Replacement, Refinement, and Reduction) principle and in accordance with the provisions of the Institutional Animal Care and Use Committee of Zunyi Medical University.

### 2.2. Isolation and Culture of PBMSCs and Preparation of PBMSC-Conditioned Media (P-CM)

Isolation and cultivation of rat PBMSCs were carried out as previously described [27]. Briefly, specific pathogen-free SD rats were intraperitoneally injected with a granulocyte colony stimulating factor (100 *μ*g/kg/day; Qilu Pharmaceutical Co., Ltd, Shandong, China) for 5 days. Following these injections, rats were anesthetized intraperitoneally under aseptic conditions, and approximately 5 mL peripheral blood was drawn from the left ventricle. Subsequently, blood samples were diluted with the same volume of phosphate-buffered saline (PBS) and slowly overlaid onto a Ficoll lymphocyte isolation solution (1.083 g/L, Tianjin Haoyang Biological Products Technology Co., Ltd., Tianjin, China). After centrifugation at 400 × *g* for 20 min, cells at the interface were collected and resuspended in 3 mL *α*-Eagle's Minimum Essential Medium (*α*-MEM, Gibco, New York, USA) containing 10% fetal bovine serum (FBS, Gibco), 2 mM glutamine (Amresco, Ohio, USA), and 0.01 *μ*g/mL basic fibroblast growth factor (bFGF; Peprotech, Rocky Hill, New Jersey, USA). The target cells were cultured in an incubator at 37°C and 5% CO_2_ with saturated humidity and subcultured at 1 : 3 with 80% confluency. Passage 3 (P3) cells were used for subsequent identification and coculture experiments. The P3 supernatant was collected and filtered with a 0.25 *μ*m needle filter to prepare P-CM, which was then stored at -80°C until subsequent experiments. Mesoderm multilineage differentiation capacity experiments were carried out in accordance with our previous research [27].

### 2.3. Isolation and Culture of M0 Macrophages

Preparation of L929 conditioned media (LCM) and isolation and culture of bone marrow-derived macrophages were performed as previously described [38]. Briefly, L929 cells were plated at a density of 1.0 × 10^5^ cells/mL in a 75 cm^2^ flask containing 55 mL Dulbecco's Modified Eagle Medium (Gibco) supplemented with 10% FBS. Subsequently, cells were incubated at 37°C and 5% CO_2_ under saturated humidity. After 7 days, the supernatant was collected, filtered through a 0.25 *μ*m needle filter, and then divided into 50 mL centrifuge tubes for subsequently culturing macrophages. SD rats were sacrificed after anesthesia, and femurs were separated on an ultraclean platform to expose the marrow cavities. Roswell Park Memorial Institute-1640 (RPMI-1640; Gibco) media containing 2% FBS was used to rinse the bone marrow cavities to make a single-cell suspension (using 1 mL disposable sterile syringes). RPMI-1640 supplemented with 10% FBS and 20% LCM (defined as M-CM) was used to resuspend the cells after the supernatant was removed by centrifugation at 400 × *g*. Subsequently, the suspension was seeded into 6-well plates and cultured in an incubator at 37°C with 5% CO_2_. After 3 days, the media was changed for the first time. Cells harvested after 6 days of cultivation were considered M0 macrophages and were identified by flow cytometry.

### 2.4. Indirect Coculture of PBMSCs and M0 Macrophages

The indirect coculture system of macrophages and PBMSCs was established using the Transwell system (0.4 *μ*m). Cultures were divided into 5 groups: PBMSC; M0; P-CM+M-CM; P-CM+M0; and PBMSC+M0. In the M0, P-CM+M0, and PBMSC+M0 groups, bone marrow-derived macrophages were plated in the lower chamber at a density of 1.0 × 10^6^/cm^2^, and 1 mL RPMI-1640 (with 10% FBS and 20% LCM) was then added. In the PBMSC and the PBMSC+M0 groups, P3 PBMSCs were seeded in the upper chamber at a density of 1.0 × 10^5^/cm^2^, and 1 mL *α*-MEM (with 10% FBS, 2 mM glutamine, and 0.01 *μ*g/mL bFGF) was then added. The upper chamber of the P-CM+M0 group was supplemented with 1 mL P-CM, the upper chamber of the M0 group was supplemented with 1 mL *α*-MEM (with 10% FBS, 2 mM glutamine, and 0.01 *μ*g/mL bFGF), and the lower chamber of the PBMSCs group was supplemented with 1 mL RPMI-1640 (with 10% FBS and 20% LCM). The P-CM+M-CM group was composed of PBMSC-conditioned media mixed with M0 macrophage-conditioned media, used for the subsequent comparison of cytokines in each group by enzyme-linked immunosorbent assay (ELISA). In addition, the M0, P-CM+M0, and PBMSC+M0 groups were further divided into three subgroups: control group, Ab9969 subgroup (1 *μ*L), and Stattic subgroup (0.1 *μ*M). After 6 h, 2 d, and 4 d of coculturing, 1 mL supernatant was collected from each group for ELISA analysis, and the adherent M0 macrophages were digested by trypsin (0.25%) for real-time quantitative polymerase chain reaction (RT-qPCR) analysis. Cells collected on day 2 were used for subsequent western blotting, immunofluorescence, and flow cytometry analysis.

### 2.5. Flow Cytometry

Target cells were adjusted to a density of 1.0 × 10^6^/mL, and 100 *μ*L of cell suspension was transferred into flow tubes. The primary antibodies used were as follows: mouse anti-rat CD11b (BD, New Jersey, USA) and CD68 (Santa Cruz, Dallas, Texas, USA), and mouse IgA (BD) and IgG2b (BD), and were used for the identification of M0 macrophages; mouse anti-rat CD29 (eBioscience, San Diego, California, USA), CD90 (eBioscience), CD44 (Santa Cruz), CD79a (Santa Cruz), CD45 (eBioscience), and CD11b (BD) were used for the identification of PBMSCs; and mouse anti-rat CD206 (Santa Cruz) and CD86 (Santa Cruz), and mouse IgG1 (BD) and IgG2b (BD) were used for the identification of M1 and M2 macrophages. After 30 min of incubation with primary antibodies, cells were subsequently incubated with Alexa Fluor-conjugated rabbit or goat anti-mouse IgG secondary antibodies. After washing with PBS and fixing with 200 *μ*L 4% paraformaldehyde, cell samples were analyzed by FACSCalibur flow cytometry (Becton Dickinson, USA), and data were analyzed using Cell Quest software.

### 2.6. Immunocytochemistry

After adjusting the cell density to 1.0 × 10^5^/cm^2^, P3 PBMSCs were plated into 6 well plates and cultured until approximately 80% confluent. Mouse anti-rat CD34 (Santa Cruz), CD73 (Invitrogen, USA), and CD105 (Invitrogen), and rabbit anti-rat HLA-DR (Abcam, Cambridge, UK) primary antibodies were added and incubated with the cells overnight at 4°C. After washing thrice with PBS, goat anti-rabbit secondary antibodies (Invitrogen) were added and incubated with the cells for 30 min at 37°C. Following this, DAB color development (Sigma–Aldrich; Merck KGaA) and hematoxylin redyeing (Solarbio Science & Technology) were conducted according to manufacturer's instructions, and the samples were then observed and analyzed under a light microscope.

### 2.7. ELISA

After 6 h, 2 d, and 4 d of cultivation, the supernatants from each group were collected and analyzed using rat IL-10, IL-1*β*, and TNF-*α* ELISA kits (Wuhan Enzyme Immunobiotech Co., Ltd., Wuhan, China) according to the manufacturer's instructions.

### 2.8. RT-qPCR

The mRNA levels of IL-10, IL-1*β*, and TNF-*α* in M0 macrophages were analyzed by RT-qPCR at 6 h, 2 d, and 4 d. Total mRNA was extracted using Trizol (TaKaRa, Japan) according to the manufacturer's protocol. Reverse transcription was carried out using the PrimeScript RT Reagent Kit (TaKaRa), with reaction conditions set to 37°C for 15 min, followed by 85°C for 5 s. Subsequent DNA amplification was performed using a SYBR Premix Extaq™ II (TaKaRa), with reaction conditions set to 95°C predenaturation for 30 s and then 40 cycles of 95°C for 5 s and 60°C for 30 s. The relative mRNA expression levels were analyzed using the 2^-*ΔΔ*Ct^ method.

### 2.9. Western Blots

M0 macrophages collected from each group after 2 d were lysed with radioimmunoprecipitation assay lysis buffer (Beijing Solarbio Science & Technology Co., Ltd., Beijing, China) containing 1% phenylmethylsulfonyl fluoride (Beijing Solarbio Science & Technology Co., Ltd.) and 1% phosphatase inhibitor (Beijing Solarbio Science & Technology Co., Ltd.). Cell lysates were then centrifuged at 12000 × *g* for 5 min, and the supernatant, containing soluble proteins, was collected. The total protein concentration was determined using a bicinchoninic acid protein assay kit (Beyotime Biotechnology, Jiangsu, China). Equal amounts of proteins were separated by sodium dodecyl sulfate-polyacrylamide gel electrophoresis and transferred to polyvinylidene difluoride membranes (Millipore, USA). After blocking with 5% bovine serum albumin (BSA, Beijing Solarbio Science & Technology Co., Ltd.) at room temperature for 1 h, the membranes were incubated with primary antibodies (all obtained from Wuhan Sanying Biological Technology Co., Ltd., Wuhan, China) against JAK1 (1 : 1000), p-JAK1 (1 : 1000), STAT3 (1 : 1000), p-STAT3 (1 : 1000), and *β*-actin (1 : 1000) overnight at 4°C. This was followed by incubation with horse radish peroxidase-conjugated goat anti-rabbit secondary antibody (1 : 1000) at room temperature for 1 h. After visualization with an enhanced chemiluminescence kit (Millipore) according to the manufacturer's instructions, protein bands were measured and analyzed using a gel imaging analyzer (Bio-Rad, USA) and ImageJ software, respectively.

### 2.10. Immunofluorescence (If)

The M0 macrophages cultured in 6-well plates were fixed with 4% paraformaldehyde for 15 min, permeabilized with 0.3% Triton X-100 for 20 min, and then blocked with BSA at room temperature for 30 min. Thereafter, samples were incubated with rabbit anti-rat STAT3 primary antibody (1 : 1000, Proteintech Group, Inc., Wuhan, China) overnight at 4°C. After 3 washes with PBST, cells were incubated with Alexa Fluor-conjugated goat anti-rabbit secondary antibody (1 : 1000, Invitrogen) at room temperature for 1 h, avoiding light. After further washing with PBST, nuclei were stained with 4′,6-diamidino-2-phenylindole for 5 min, in the dark. The fluorescent signals were analyzed using an inverted fluorescence microscope (Olympus Corporation, Tokyo, Japan).

### 2.11. Statistical Analysis

All data were processed and analyzed using SPSS 18.0 software and presented as the mean ± standard deviation. One-way analysis of variance was used to compare the means of multiple groups. Differences were considered statistically significant at *P* < 0.05.

## 3. Results

### 3.1. Identification of PBMSCs and Macrophages

**Figure 1:**
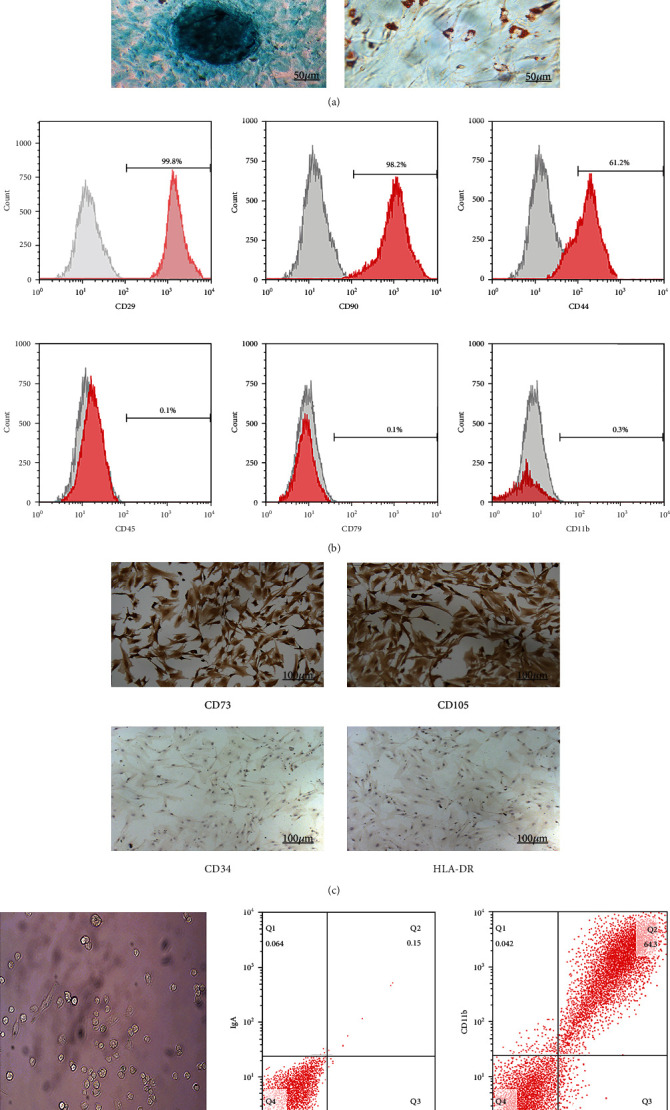
Characterization of PBMSC morphology, multidirectional differentiation potential, and phenotype and of macrophage morphology and phenotype. (a) Growth morphology of PBMSCs cultured for 24 h (×100). After induction of osteoblasts, chondroblasts, and adipoblasts for 21 days, the differentiation ability of PBMSCs was evaluated using Alizarin Red, Alcian blue, and oil red O staining, respectively. (b) P3 PBMSCs were positive for CD29, CD90, and CD44, but negative for CD45, CD79, and CD11b. (c) Hematoxylin-stained P3 PBMSCs were positive for CD73 and CD105 expression, but negative for CD34 and HLA-DR expression. (d) Left: morphology of M0 macrophages after 10 days of culture (×100). Right: expression of CD11b, CD68, IgA, and IgG2b in M0 macrophages. CD: cluster of differentiation; PBMSCs: peripheral blood-derived mesenchymal stem cells.

After 24 h of primary culture, isolated MSCs showed fusiform and adherent growth phenotypes; after 21 days of induction by the corresponding medium, the cells were able to differentiate into osteocytes, chondrocytes, and adipocytes, as identified by staining (Figure 1(a)). Flow cytometry and immunocytochemistry analysis identified that the cultured cells highly expressed CD29, CD90, CD44, CD73, and CD105, but did not express CD45, CD79, CD11b, CD34, and HLA-DR (Figures 1(b) and 1(c)). After 10 days of culturing, macrophages harvested from rat bone marrow were observed to grow adherently and were round in shape, with the extension of pseudopodia; flow cytometry analysis revealed that CD11b and CD68 were highly expressed in these cells (Figure 1(d)).

### 3.2. Effects of PBMSCs on the Expression and Secretion of Inflammatory Cytokines by Macrophages

**Figure 2:**
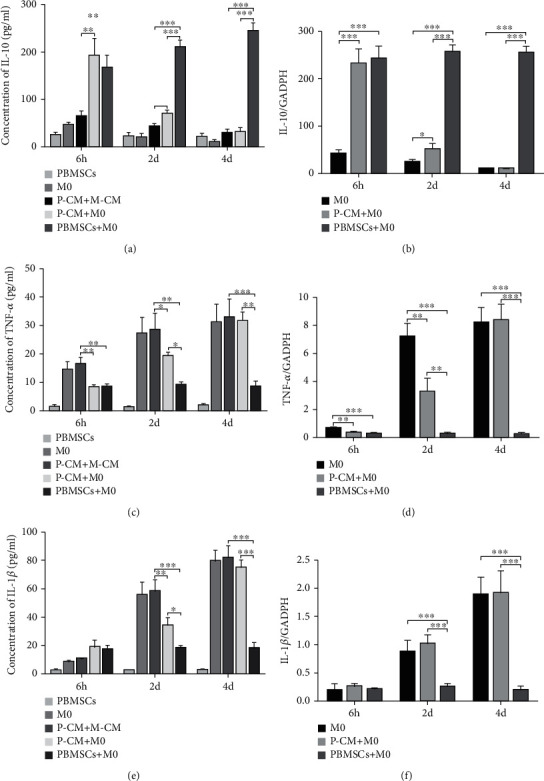
Effect of various coculturing conditions on the secretion and expression of inflammatory cytokines by macrophages. (a, c, e) Concentrations of IL-10 (a), TNF-*α* (c), and IL-1*β* (e) in the supernatant of various coculture groups were measured by ELISA at 6 h, 2 d, and 4 d. (b, d, f) The expression levels of IL-10 (b), TNF-*α* (d), and IL-1*β* (f) in macrophages from various coculture groups were measured by RT-qPCR at 6 h, 2 d, and 4 d. Graphs show the mean ± standard deviation; *n* = 3; ^∗^*P* < 0.05,  ^∗∗^*P* < 0.01, and^∗∗∗^*P* < 0.001. ELISA: enzyme-linked immunosorbent assay; GAPDH: glyceraldehyde 3-phosphate dehydrogenase; IL: interleukin; M-CM: Roswell Park Memorial Institute-1640 media with 10% fetal bovine serum and 20% L929 conditioned media; P-CM: PBMSC-conditioned media; PBMSCs: peripheral blood-derived mesenchymal stem cells; RT-qPCR: real-time quantitative polymerase chain reaction; TNF: tumor necrosis factor.

The expression and secretion of inflammatory factors by macrophages from each coculture group were analyzed by ELISA and RT-qPCR, respectively. IL-10, an important anti-inflammatory cytokine, had significantly higher levels of secreted proteins and mRNA expression at 2 d and 4 d in the PBMSC+M0 coculture group compared with those in the P-CM+M0 group (Figures 2(a) and 2(b), *P* < 0.01 or *P* < 0.001). This was opposite to the changes seen in the pronflammatory cytokine, TNF-*α*, at the same time points (Figures 2(c) and 2(d), *P* < 0.01 or *P* < 0.001). While IL-1*β* had a slightly elevated expression in macrophages after coculturing with PBMSCs for 6 h (Figure 2(f), *P* < 0.05), the amount of IL-1*β* in the supernatant did not show a significant difference between the PM-CM+M-CM and the PBMSC+M0 groups (Figure 2(e), *P* > 0.05) and its expression and secretion significantly decreased on days 2 and 4 (Figures 2(e) and 2(f), *P* < 0.001). We also compared the P-CM+M0 group to the PM-CM+M-CM groups. Here, the secretion of IL-10 increased, while those of TNF-*α* decreased, in the P-CM+M0 group compared to the PM-CM+M-CM groups at 6 h and 2 d (Figures 2(a) and 2(c), *P* < 0.05 or *P* < 0.01). The expression of IL-1*β* increased at 6 h in the P-CM+M0 group compared to the M0 group (Figure 2(f), *P* < 0.05), but there was no significant difference in secretion between this two groups (Figure 2(e), *P* > 0.05), similar to the differences between the PBMSC+M0 and M0/P-CM+M-CM groups at the same time points. On day 4, there was no significant difference in the expression and secretion of IL-10, TNF-*α*, and IL-1*β* in the P-CM+M0 group compared with those in the M0 group (Figures 2(a)–2(f), *P* > 0.05). When comparing the PBMSC+M0 group to the P-CM+M0 group, the expression and secretion of IL-10, TNF-*α*, and IL-1*β* did not show significant differences at 6 h (Figure 2(a)–2(f), *P* > 0.05). On days 2 and 4, IL-10 increased (Figure 2(a), 2(b), *P* < 0.001), and TNF-*α* and IL-1*β* decreased (Figure 2(c)–2(f), *P* < 0.05, *P* < 0.01, or *P* < 0.001), in the PBMSC+M0 group compared with those in the P-CM+M0 group. These results indicate that both PBMSCs and their conditioned media promoted the expression and secretion of IL-10 in macrophages and inhibited the expression and secretion of TNF-*α* and IL-1*β*. Furthermore, they indicate that the capacity of PBMSCs to regulate the expression and secretion of macrophage inflammatory cytokines was stronger than that of the conditioned media.

### 3.3. Influence of Different Coculture Times on the Secretion and Expression of Inflammatory Cytokines by Macrophages

**Figure 3:**
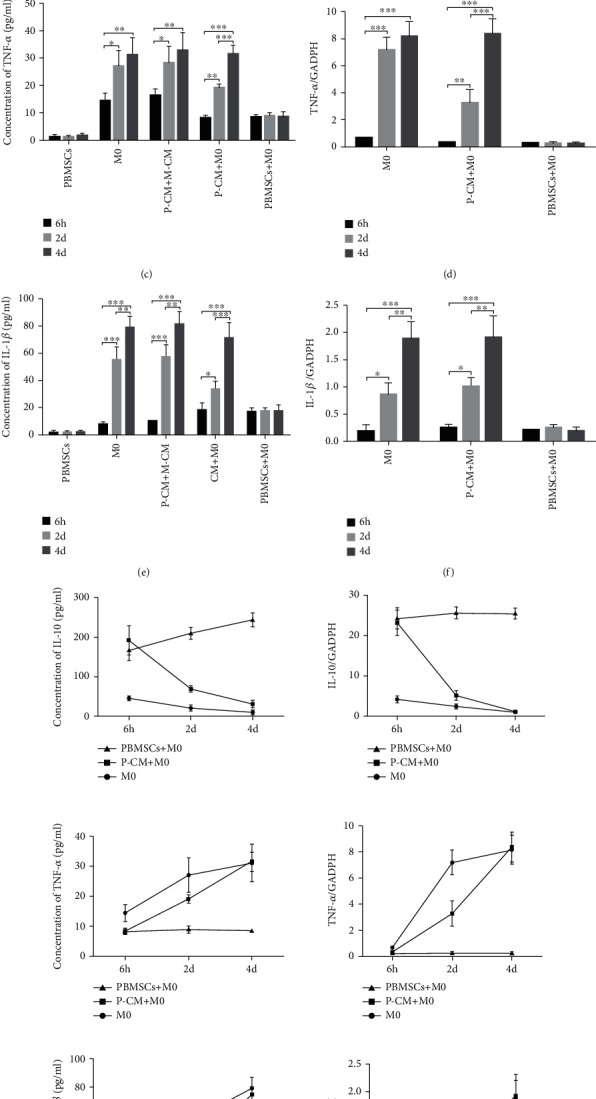
Influence of different coculture times on the secretion and expression of inflammatory cytokines by macrophages. (a, c, e) Concentrations of IL-10 (a), TNF-*α* (c), and IL-1*β* (e) in the supernatant of each group were determined by ELISA at 6 h, 2 d, and 4 d. (b, d, f) The mRNA expression levels of IL-10 (b), TNF-*α* (d), and IL-1*β* (f) from macrophages of the M0, P-CM+M0, and PBMSC+M0 groups were detected by RT-qPCR at 6 h, 2 d, and 4 d. (g) The secretion (left) and expression (right) of inflammatory cytokines at different time points for various culture groups. Graphs show the mean ± standard deviation; *n* = 3; ^∗^*P* < 0.05,  ^∗∗^*P* < 0.01, and^∗∗∗^*P* < 0.001. ELISA: enzyme-linked immunosorbent assay; GAPDH: glyceraldehyde 3-phosphate dehydrogenase; IL: interleukin; M-CM: Roswell Park Memorial Institute-1640 media with 10% fetal bovine serum and 20% L929 conditioned media; P-CM: PBMSC-conditioned media; PBMSCs: peripheral blood-derived mesenchymal stem cells; RT-qPCR: real-time quantitative polymerase chain reaction; TNF: tumor necrosis factor.

From the ELISA and RT-qPCR results, the effects of various coculture times on macrophage inflammatory cytokine expression and secretion were also analyzed. In the M0-only group, the secretion and expression of IL-10 gradually decreased at the three successive culture time points of 6 h, 2 d, and 4 d (Figures 3(a), 3(b), and 3(g); *P* < 0.05, *P* < 0.01, or *P* < 0.001), while TNF-*α* and IL-1*β* exhibited the opposite trend (Figures 3(c)–3(g); *P* < 0.05, *P* < 0.01, or *P* < 0.001); however, there was no significant difference in the expression and secretion of TNF-*α* between days 2 and 4 (Figures 3(c) and 3(d), *P* > 0.05). In the P-CM+M0 group, the secretion and expression of IL-10 decreased successively at the three coculture time points of 6 h, 2 d, and 4 d (Figures 3(a), 3(b), and 3(g); *P* < 0.05, *P* < 0.01, or *P* < 0.001), while TNF-*α* and IL-1*β* levels increased progressively (Figures 3(c)–3(g); *P* < 0.05, *P* < 0.01, or *P* < 0.001). In the PBMSC+M0 group, ELISA analysis identified that the amount of IL-10 in the supernatant increased at 2 and 4 d compared with that at 6 h (Figure 3(a); *P* < 0.05 or *P* < 0.01), but there was no significant difference in IL-10 secretion between days 2 and 4 (Figure 3(a), *P* > 0.05). RT-qPCR analysis indicated that the mRNA expression levels of IL-10 increased, but did not show significant differences between the three time points (Figures 3(b) and 3(g), *P* > 0.05). In this group, both the secretion and expression of TNF-*α* and IL-1*β* decreased compared with those in other groups, but there was no significant difference between the three time points (Figures 3(c)–3(g), *P* > 0.05). These results suggest that the ability of PBMSCs to regulate the expression and release of inflammatory cytokines by macrophages was maintained at a high level, while the regulatory effects of P-CM decreased over time.

### 3.4. Effect of the Inhibition of the IL-10/STAT3 Pathway on the Secretion and Expression of Inflammatory Cytokines

**Figure 4:**
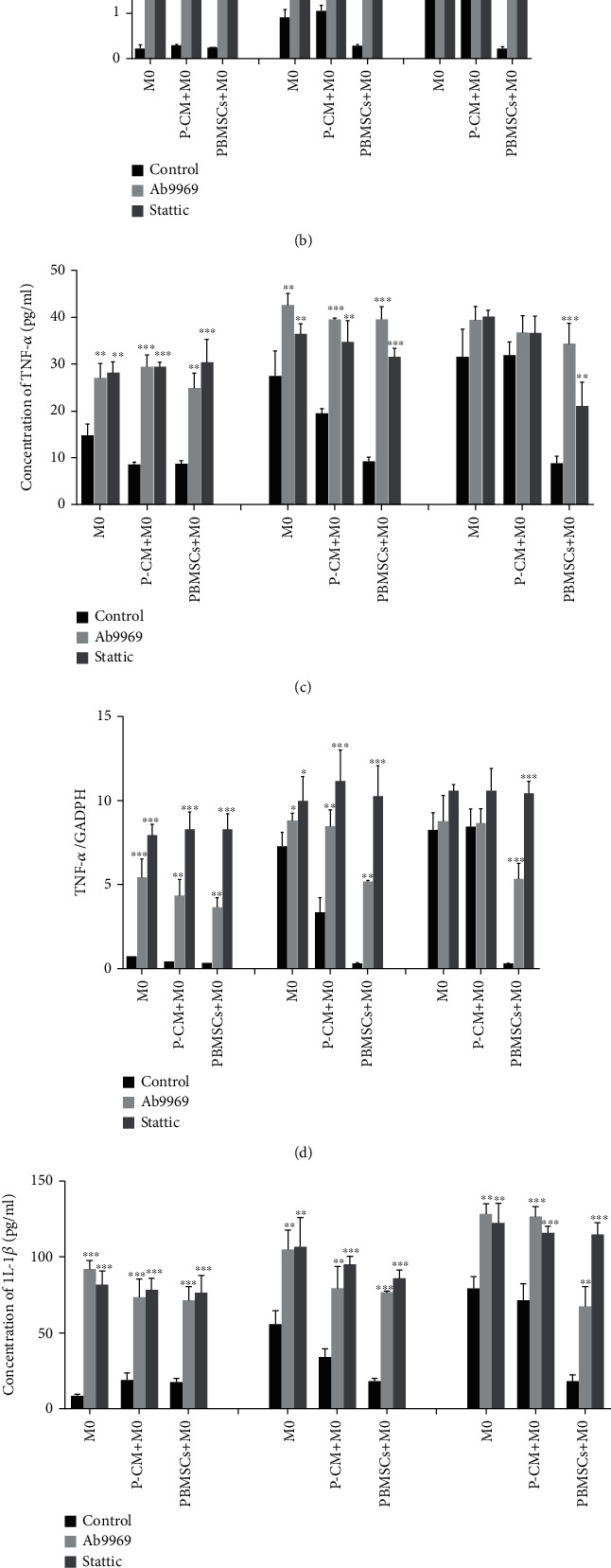
Effect of inhibiting the IL-10/STAT3 pathway on the secretion and expression of inflammatory cytokines in various groups. (a, c, e) Concentrations of IL-10 (a), TNF-*α* (c), and IL-1*β* (e) in the supernatant from the M0, P-CM+M0, and PBMSC+M0 groups were detected by ELISA, after inhibiting the IL-10/STAT3 pathway for 6 h, 2 d, or 4 d. (b, d, f) mRNA expression levels of IL-10 (b), TNF-*α* (d), and IL-1*β* (f) in macrophages from the M0, P-CM+M0, and PBMSC+M0 groups were measured by RT-qPCR after inhibiting the IL-10/STAT3 pathway for 6 h, 2 d, or 4 d. Graphs show the mean ± standard deviation; *n* = 3; ^∗^*P* < 0.05,  ^∗∗^*P* < 0.01, and^∗∗∗^*P* < 0.001, compared with the M0 group. ELISA: enzyme-linked immunosorbent assay; GAPDH: glyceraldehyde 3-phosphate dehydrogenase; IL: interleukin; P-CM: PBMSC-conditioned media; PBMSCs: peripheral blood-derived mesenchymal stem cells; RT-qPCR: real-time quantitative polymerase chain reaction; TNF: tumor necrosis factor.

To verify whether the IL-10/STAT3 pathway can regulate the secretion and expression of IL-10, IL-1*β*, and TNF-*α*; Ab9969 and Stattic were applied immediately after cocultures were set up. ELISA and RT-qPCR analysis demonstrated that the administration of Ab9969 or Stattic dramatically inhibited the secretion and expression of IL-10 at 6 h, 2 d, and 4 d (Figures 4(a) and 4(b); *P* < 0.05, *P* < 0.01, or *P* < 0.001), whereas IL-1*β* showed the opposite secretion and expression trends at the same time points (Figures 4(e) and 4(f); *P* < 0.05, *P* < 0.01, or *P* < 0.001). Moreover, the secretion and expression of TNF-*α* increased at 6 h and 2 d (Figures 4(c) and 4(d); *P* < 0.05, *P* < 0.01, or *P* < 0.001); however, there was no significant effect of these treatments on the secretion and expression of TNF-*α* by day 4, except in the PBMSC+M0 group (Figures 4(c) and 4(d); *P* > 0.05). These results indicate that the activation of the IL-10/STAT3 pathway not only upregulated the expression and secretion of IL-10 but also inhibited the expression and secretion of IL-1*β* and TNF-*α*, suggesting, to some extent, that this pathway is involved in regulating the balance of M1/M2 macrophages.

### 3.5. Effect of PBMSCs on the IL-10/STAT3 Pathway in Macrophages

**Figure 5:**
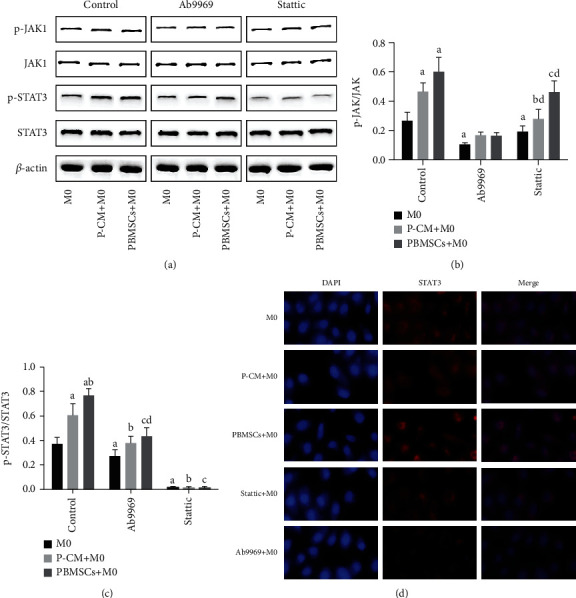
Phosphorylation of JAK1 and STAT3 proteins in various culture groups. (a) WB results of JAK1/p-JAK1, STAT3/p-STAT3, and *β*-actin. (b) Phosphorylation states of JAK1 in various culture groups. (c) Phosphorylation states of STAT3 in various culture groups. (d) Intracellular distribution of STAT3 (red) in macrophages; DAPI: nuclear staining (blue). Graphs show the mean ± standard deviation; *n* = 3; ^a^*P* < 0.05 compared with M0 group in the control group, ^b^*P* < 0.05 compared with the P-CM+M0 group in the control group, ^c^*P* < 0.05 compared with the PBMSC+M0 group in the control group, ^d^*P* < 0.05 compared with M0 group in the Ab9969 group, and ^e^*P* < 0.05 compared with the M0 group in the Stattic group. JAK: Janus kinases; PBMSCs: peripheral blood-derived mesenchymal stem cells; STAT3: Signal transducer and activator of transcription 3; P-CM: PBMSC-conditioned media; DAPI: 4′,6-diamidino-2-phenylindole.

Given that IL-10 secreted by PBMSCs may mediate macrophage polarization through the activation of the JAK1/STAT3 pathway, western blotting was used to measure the expression of JAK1/p-JAK1 and STAT3/p-STAT3 proteins. As shown in Figure 5, compared with the M0 macrophage-only group, the expression of p-JAK1 and p-STAT3 significantly increased in the coculture group after 2 days of indirect coculture; the expression of p-STAT3 in the PBMSC+M0 coculture group was higher than that in the P-CM+M0 group (*P* < 0.05), although there was no significant difference in the expression of JAK1 between these two groups (*P* > 0.05). The addition of Ab9969 significantly decreased the expression of p-STAT3 in each group (*P* < 0.05); interestingly, under this condition, the expression of p-JAK1 was higher in the coculture groups than in the M0 macrophage-only group, and the expression of p-STAT3 in the PBMSC+M0 group was higher than that in the M0/P-CM+M0 groups. Moreover, after the addition of Stattic, the expression of p-STAT3 significantly decreased in the coculture group compared that in the corresponding control groups (*P* < 0.05). Activated STAT3 dimerizes and translocates into the nucleus to initiate transcription [39, 40]. Therefore, in the current study, we also used immunofluorescence to further verify whether IL-10 secreted by PBMSCs could promote the nuclear translocation of phosphorylated STAT3. As shown in Figure 5(d), in the PBMSC+M0 group, STAT3 was mainly located in the nucleus, as indicated by the red staining. However, the levels of STAT3 nuclear translocation were significantly downregulated after treatment with the inhibitors Ab9969 or Stattic. Overall, these results suggest that IL-10 secreted by PBMSCs mediated macrophage polarization towards the M2 phenotype by promoting JAK1/STAT3 activation and the nuclear translocation of STAT3.

### 3.6. Effect of PBMSCs and IL-10/STAT3 Pathway on the Polarization of Macrophages

**Figure 6:**
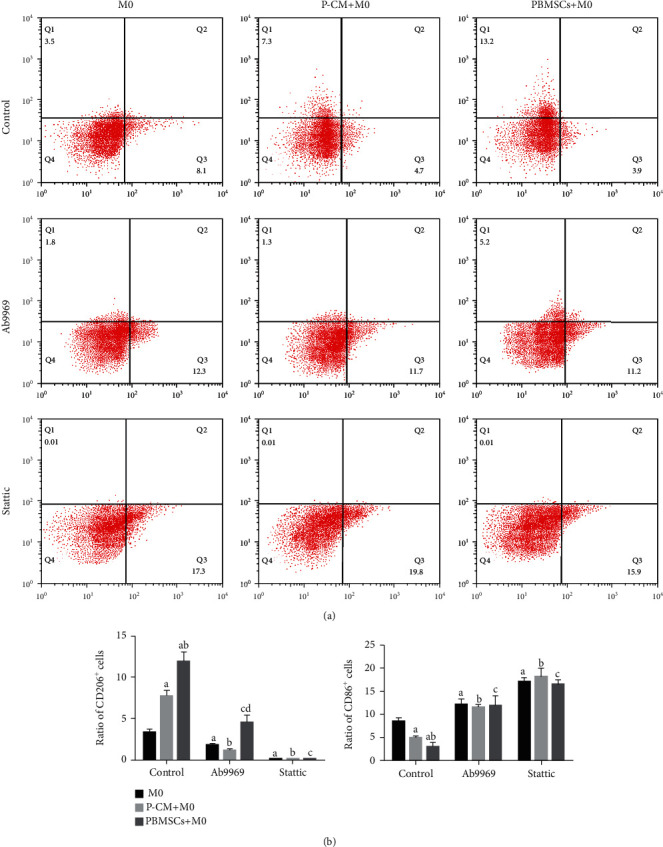
Phenotypes of macrophages from various groups as detected by flow cytometry. (a) The expression of CD206 and CD86 in macrophages under different culture conditions. (b) The ratio of CD206^+^ or CD86^+^ cells in various groups. Graphs show the mean ± standard deviation; *n* = 3; ^a^*P* < 0.05 comparing the M0 group vs. the control group, ^b^*P* < 0.05 comparing the P-CM+M0 group vs. the control group, ^c^*P* < 0.05 comparing the PBMSC+M0 group vs. the control group, and ^d^*P* < 0.05 comparing the M0 group vs. the Ab9969 group. CD: cluster of differentiation; P-CM: PBMSC-conditioned media; PBMSCs: peripheral blood-derived mesenchymal stem cells.

As CD206 and CD86 have been demonstrated to be specific hallmarks of M2 and M1 macrophages, respectively [41, 42], we evaluated the expression of these proteins to determine the polarization of macrophages. As shown in Figure 6, flow cytometry analysis demonstrated that compared with the M0-only group, the expression of CD86 decreased on day 2, while the expression of CD206 significantly increased in the coculture group; the proportion of CD206 positive cells in the PBMSC+M0 group was higher than that in the P-CM+M0 group. After treatment with the inhibitors Ab9969 or Stattic, the proportion of CD206-positive cells in each group remarkably decreased compared with that in the corresponding control group (*P* < 0.05), and the proportion of CD86 positive cells significantly increased (*P* < 0.05); moreover, in the Ab9969 group, the proportion of CD206 positive cells in the PBMSC+M0 group was higher than that in the other two groups (*P* < 0.05). These results suggest that PBMSCs are able to upregulate the M2/M1 ratio by the activation of the IL-10/STAT3 pathway in macrophages.

## 4. Discussion

Macrophages are regarded as one of the most important cell types in the inflammatory microenvironment due to their extensive functional plasticity, which involves roles beyond pathogen defense and immune functions [43, 44]. In response to specific signals at a wound site, a shift from proinflammatory M1 to anti-inflammatory M2 macrophages is critical for tissue remodeling [45]. Normally, a clear functional shift of macrophages occurs during skin and muscle wound healing [46–48]. Early on, M1 macrophages play a predominant role in removing weakened neutrophils and destroying pathogens in wounds, creating a sterile environment for subsequent tissue repair [49, 50]. Conversely, in the later stages of inflammation, owing to the transformation of signals from the microenvironment, macrophages are reprogrammed to the M2 phenotype, which promotes the regression of inflammation and functional recovery through the release of anti-inflammatory cytokines and induction of matrix-degrading enzymes [49–51]. However, studies involving various inflammatory diseases, especially SCI, have shown that proinflammatory M1 macrophages predominate and persist for a long time at the injury site, and an M1 to M2 macrophage shift cannot be observed, resulting in a chronic inflammatory period post-injury [52–54]. This severely unbalanced and persistent macrophage response occurs in many inflammatory diseases, and such a response always exacerbates the associated disease. Hence, in order to improve the prognosis of inflammatory diseases, it is imperative to induce macrophages to switch towards the M2 phenotype [55–57].

Over the past decade, numerous studies have shown that MSC transplantation could serve as a promising therapeutic approach for many inflammatory diseases [24, 58]. Research has shown that after SCI, MSC transplantation can improve functional and anatomical recovery, associated with the induction of a functionally and phenotypically heterogeneous macrophage response [59–61]. Similarly, one study on MSC transplantation in a mouse model of peritonitis demonstrated the vase reprogramming of inflammatory macrophages towards the M2 phenotype, in parallel with the correction of disease parameters [62]. Furthermore, peritoneal macrophages cocultured with MSCs in vitro could also be greatly induced towards the M2 phenotype, accompanied by an increase in anti-inflammatory cytokines such as IL-10, and this polarized M2 transplantation was also able to reduce the systemic inflammatory response induced by LPS [63, 64]. All studies mentioned above have shown that MSC transplantation can improve the prognosis of inflammatory diseases, mainly by inducing the polarization of macrophages towards the anti-inflammatory M2 phenotype [65]. However, to date, the specific mechanism of MSC-mediated M2 macrophage polarization is still poorly understood.

MSCs are known to secrete various anti-inflammatory cytokines, such as IL-10 and TGF-*β*, which are directly or indirectly involved in immunomodulatory effects [66]. Previously, we built a Transwell coculture system of PBMSCs and macrophages and found that compared to other cytokines such as IL-6, TGF-*β*, and IFN-*γ*, the levels of IL-10 in the supernatant were significantly higher in this coculture system [67]. IL-10, an important anti-inflammatory cytokine, plays a key regulatory role in the growth, proliferation, metabolism, and phenotypic shift of macrophages [32]. Moreover, studies have demonstrated that the JAK1/STAT3 pathway is a significant downstream pathway for IL-10-mediated M2 macrophage polarization [34, 35]. Accordingly, we speculated that IL-10 secreted by PBMSCs could promote a series of M2-associated gene expression changes via the activation of STAT3, resulting in the secretion of anti-inflammatory cytokines and reduced levels of proinflammatory cytokines, thus promoting tissue repair and inducing a functional improvement against inflammatory diseases.

In the present study, we found that macrophages cocultured with PBMSCs and their conditioned media (P-CM) exhibited similar cytokine expression and secretion patterns to M2 macrophages, showing significant reductions in the levels of IL-1*β* and TNF-*α*, and significantly upregulating the levels of IL-10. However, at 6 h, RT-qPCR revealed that the mRNA levels of IL-1*β* in macrophages in the coculture group were higher than those in the M0-only group; conversely, ELISA analysis showed that the amount of IL-1*β* in the supernatant was not significantly different between groups. This finding may be associated with the activation of macrophage Toll-like receptors. Studies have documented that Toll-like receptors can bind to damage-associated molecular patterns, such as nucleic acid fragments released by dead PBMSCs, thus stimulating the expression of IL-1*β*-associated genes and synthesizing its precursor (Pro-IL1*β*). However, Pro-IL1*β* is unable to mature and is not secreted out of the cell unless cleaved by an activated caspase, which can be realized after the activation of intracellular inflammasomes [68, 69]; moreover, it has been shown that after activation by extracellular IL-10, phosphorylated STAT3 can inhibit the activation of inflammasomes via the mTOR receptor [33]. Therefore, although the mRNA levels of IL-1*β* were elevated in macrophages, the precursor could not be cleaved, resulting in unaltered or even reduced levels of IL-1*β* secretion; this further confirms, from another perspective, the activation of STAT3 in macrophages from the coculture group. The analysis of IL-10 identified that it can promote its own expression after activating macrophage STAT3, indicating positive feedback regulation; paradoxically, a sustained decrease in the expression and secretion of IL-10 was observed over time in the P-CM+M0 coculture group. This interesting phenomenon may be related not only to the degradation of IL-10 but also to the activation of suppressors of cytokine signaling (SOCS; JAK/STAT3 pathway inhibitory proteins), thus creating negative feedback regulation [70]. In contrast, the expression and secretion of IL-10 in the PBMSC+M0 group showed a positive feedback, and we suspect that this divergent effect might be due to the inhibition of SOCS by PBMSCs in some way, which remains to be clarified. Moreover, both Ab9969 and Stattic could antagonize the anti-inflammatory effect of macrophages cocultured with PBMSCs and P-CM, indicating that IL-10 and the activation of STAT3 in the coculture system could regulate the expression and secretion of cytokine characteristic of M1/M2 macrophages.

Furthermore, through the analysis of the expression and secretion of cytokines at different time points in each group, it was found that P-CM and PBMSCs had an analogous ability to regulate inflammatory cytokines within 6 h. However, compared with the 6 h time point, the expression and secretion of IL-1*β* and TNF-*α* increased at day 2 in the P-CM+M0 group, accompanied by decreased levels of IL-10, and at day 4, the levels of all inflammatory cytokines were equivalent to those in the M0 group. This indicates that the regulatory ability of P-CM gradually decreased, which could be explained by the degradation of anti-inflammatory cytokines in the P-CM+M0 group over time. In contrast, in the PBMSC+M0 coculture group, the amount of IL-10 increased gradually until reaching a relatively stable and high level of secretion, whereas the inflammatory cytokines (TNF-*α* and IL-1*β*) were consistently maintained at a low level, indicating that the long-term regulatory effect of PBMSCs on inflammation was better than that of P-CM. There may be a long-term interaction between PBMSCs and macrophage-PBMSCs that secrete IL-10, modulating an M0-to-M2 shift via the JAK1/STAT3 pathway; in turn, macrophages also moderate the function of PBMSCs, in part through the release of inflammatory cytokines. The interaction between PBMSCs and macrophages resulted in better anti-inflammatory effects compared to the simple addition of P-CM. Still, the mechanism underlying this complex interaction needs to be further studied. Notably, when M0 macrophages were cultured alone, the expression and secretion of proinflammatory cytokines gradually increased, while the levels of anti-inflammatory cytokines gradually reduced, indicating that M0 macrophages tended to spontaneously polarize towards the proinflammatory M1 phenotype.

The ELISA and RT-qPCR results from this study showed that IL-10 secreted by PBMSCs, and the activation of STAT3 in macrophages, could promote the secretion and expression of the M2-associated cytokine IL-10 and inhibit the synthesis and release of the M1-associated cytokines TNF-*α* and IL-1*β*, preliminarily suggesting that PBMSCs could moderate the polarization of M2 macrophages through the IL-10/STAT3 pathway. We then performed western blotting, immunofluorescence, and flow cytometry to further clarify whether PBMSCs could secrete IL-10 and modulate M2 polarization via the JAK1/STAT3 pathway. We found that in the coculture group, both JAK1/STAT3 signaling in macrophages and the number of M2 macrophages were significantly upregulated, while the number of M1 macrophages significantly decreased, factors that could be reversed after the administration of Ab9969 or Stattic. These results suggest that PBMSCs can mediate the polarization of M2 macrophages through the IL-10/STAT3 pathway. Moreover, we found that the proportion of M2 macrophages was consistent with the expression level of p-STAT3. After Ab9969 addition, the proportion of M2 cells and the levels of p-STAT3 in macrophages in the PBMSC+M0 coculture group were higher than those in the other two groups, suggesting that other factors may activate the JAK1/STAT3 pathway, thereby promoting the polarization of M2 macrophages. However, in general, other factors did not exert as strong a regulatory effect on macrophages as IL-10.

Taken together, the present study revealed that PBMSCs or P-CM could skew the balance of M1/M2 polarization to anti-inflammatory, tissue remodeling, M2 cell types based on the activation of the IL-10/STAT3 pathway. Multiple studies, including our previous work, have shown that MSC transplantation in vivo could significantly improve the outcome of various inflammatory diseases, with an increase in the levels of the anti-inflammatory factor IL-10 and number of M2 macrophages in the injured area [29, 60]. Nevertheless, whether PBMSCs and P-CM transplantation in vivo could also mediate the activation of the M2 phenotype via this pathway remains to be confirmed. In addition, our experiment suggested that there was an interaction between PBMSCs and macrophages, which ultimately led to a better long-term regulatory effect of PBMSCs on inflammation than that of P-CM. In conclusion, the in vitro experiments preliminarily revealed the internal mechanism by which PBMSCs regulate macrophage polarization in vitro and thus control inflammation.

## 5. Conclusions

In summary, this study demonstrated that PBMSCs and P-CM could induce the polarization of macrophages towards the anti-inflammatory M2 phenotype by enhancing the activation of the IL-10/STAT3 signaling pathway. It also revealed that the long-term regulatory effect of PBMSCs on macrophage-mediated inflammation was better than that of P-CM. These findings provide an important theoretical and experimental basis for the clinical transformation of PBMSCs in the treatment of immune diseases, particularly inflammatory diseases.

## Data Availability

The underlying data supporting the results of our study can be found in Zunyi Medical University.
